# The Impact of Green Technology Innovation on Carbon Emissions in the Context of Carbon Neutrality in China: Evidence from Spatial Spillover and Nonlinear Effect Analysis

**DOI:** 10.3390/ijerph19020730

**Published:** 2022-01-10

**Authors:** Shihong Zeng, Gen Li, Shaomin Wu, Zhanfeng Dong

**Affiliations:** 1 College of Economics & Management, Beijing University of Technology, Beijing 100124, China; Lgn1912@emails.bjut.edu.cn; 2 Kent Business School, University of Kent, Kent, Canterbury CT2 7FS, UK; 3 Chinese Academy of Environmental Planning, Beijing 100012, China

**Keywords:** carbon emissions, green technology innovation, regional heterogeneity, spatial spillover effect

## Abstract

The Paris agreement is a unified arrangement for the global response to climate change and entered into force on 4 November 2016. Its long-term goal is to hold the global average temperature rise well below 2 °C. China is committed to achieving carbon neutrality by 2060 through various measures, one of which is green technology innovation (GTI). This paper aims to analyze the levels of GTI in 30 provinces in mainland China between 2001 and 2019. It uses the spatial econometric models and panel threshold models along with the slack based measure (SBM) and Global Malmquist-Luenberger (GML) index to analyze the spatial spillover and nonlinear effects of GTI on regional carbon emissions. The results show that GTI achieves growth every year, but the innovation efficiency was low. China’s total carbon dioxide emissions were increasing at a marginal rate, but the carbon emission intensity was declining year by year. Carbon emissions were spatially correlated and show significant positive agglomeration characteristics. The spatial spillover of GTI plays an important role in reducing carbon dioxide emissions. In the underdeveloped regions in China, this emission reduction effect was even more significant.

## 1. Introduction

Over the past few decades, China’s rapid economic development has brought about a series of side effects such as resource depletion and environmental degradation, which makes the sustainable development of China’s economy and society a huge challenge [1,2,3,4,5,6,7]. Carbon dioxide emissions (CDE) are a key factor in climate deterioration [8,9]. China, as the largest CDE producer in the world and one of the countries committed to achieving carbon neutrality and complete elimination of carbon emissions by 2060 [10], is under tremendous pressure to reduce emissions [10,11,12]. In order to curb the increase of CDE, the Chinese government has made a series of commitments and plans to promote a comprehensive shift in socio-economic development to a green development model, starting from 2021 [13].

With the severe challenges brought about by environmental degradation, green technology innovation (GTI), as a new innovative method that highlights green environmental protection, can not only achieve economic growth, traditionally driven by traditional technological innovation, but also effectively alleviate the dual pressures of energy and the environment. This is a cause of concern for governments around the world [14,15,16,17]. GTI is an important means to alleviate the internal contradictions between economic growth and environmental pollution, and has become a key factor in promoting green and sustainable development [18,19]. At present, China’s economy is in a critical period of high-quality development, and promoting GTI is an important way to realize the transformation and upgrading of China’s strategic emerging industries [20,21]. It is also an important measure to advocate the construction of ecological civilization and high-quality economic growth [22,23,24,25].

Regional heterogeneity and spatial interconnection are essential features affecting the influence of technological innovation on the ecological environment [26]. Owing to the existence of spatial interaction, the GTI or CDE of a region will have specific effects on the surrounding regions through diffusion or polarization. The GTI and CDE of different regions are therefore both interconnected and distinct. China has a vast territory, and each region has its own traits, ignoring the spatial spillover effect can cause biases in model estimation. As such, there is a need to conduct an in-depth study on the spatial spillover effects of both GTI and carbon emissions.

GTI is of great significance to promote carbon emission reduction and can help governments fully realize the strategic goal of carbon neutrality. However, so far, research on carbon emission factors has focused more on technological innovation and neglected the GTI, which is more closely related to carbon emission. Therefore, the aim of this research is to quantitatively analyze the levels of GTI and carbon emissions across China, explore the spatial distributing characteristics of GTI and carbon dioxide, based on which, an in-depth analysis of the spatial spillover and non-linear impact of GTI on regional CDE will be performed and relevant countermeasures and suggestions will be proposed.

The remainder of this article is structured as follows. Section 2 reviews the literature on carbon emissions and GTI. Section 3 explains the data sources, variable selection, and research methods. Section 4 presents the empirical results. Section 5 wraps up the paper and suggests further research.

## 2. Literature Review

As global climate change intensifies, environmental issues, together with food security, education, health, poverty, etc., have become the most pressing issues in the world [27,28]. An important factor leading to climate change is the emission of greenhouse gases [8,29], and carbon dioxide is a major component of greenhouse gases, so current concerns about carbon emissions has become a hot topic of scholars [28,30,31,32]. As the world’s largest carbon dioxide emitter [10,12], China contributed an average of 63.9% of the increase in global emissions from 2006 to 2016 [12]. As a responsible major country, China proposed that it should reach the peak of CDE by 2030 and achieve the strategic goal of carbon neutrality by 2060 [33]. Nowadays, accelerating GTI and transforming the economic development mode have become an important means to achieve carbon peaking and carbon neutrality goals, and therefore, achieve sustainable economic development [16,28]. It should be noted that managing to hold carbon emission peak and carbon neutrality does not blindly pursue energy conservation and carbon reduction. It aims to apply a green economic development model that can achieve harmony between humans and nature, while ensuring stable economic development, which is an economic model of sustainable development [34]. The green economy consists of sociopolitical and economic elements, the balance of which makes an economy sustainable [35]. In view of the current worsening ecological environment, green economy has become the economic development model pursued by all countries, and a topical research field among academia. Vukovic et al. [35] propose main principles and a methodology of the criteria evaluation for a regional green economy and combine the current state and dynamics of the green economy in evaluating and forecasting. Pociovălișteanu et al. [36] study a situation of green jobs at the European Union level and the relationship between environment and employment, by analyzing the link between employment and environmental policies. Dulal et al. [37] analyze the role of financial tools in Asian countries in the process of achieving green economy. To promote a green economy and achieve sustainable economic development, the primary issue is to control greenhouse gas emissions. Therefore, it is necessary to conduct an in-depth study on reducing carbon dioxide emissions.

GTI is a term widely used in different research areas, its goal being to achieve cleaner production, improving environmental performance, and promoting the comprehensive utilization of resources and energy [38,39,40,41]. It not only emphasizes technological innovation to save resources and energy, but also requires the reduction or even elimination of pollution and damage to the ecological environment [20]. As a new innovative method that highlights green and environmental protection, GTI can not only achieve economic growth driven by traditional technological innovation, but also alleviate the dual pressure of energy and the environment. It can therefore promote low-carbon sustainable economic development [42,43,44,45,46]. In the literature, there is much research on the mechanism and effectiveness of specific green technologies to improve environmental performance [47,48,49,50]. For example, Diaz et al. [51] analyzes the impact of carbon capture technology on carbon emission reduction in view of greenhouse gas emissions in the U.S. refining industry, and finds that carbon capture technology can effectively reduce carbon emissions. López et al. [52] find that technological innovations, such as electric buses and emission-free buses, should be prioritized in order to achieve a greater performance in the environmental dimension. Green technologies, such as carbon capture, waste management, and power generation technologies, are expected to significantly alleviate the global energy, environment, and climate change crises in the future [53,54,55].

For the measurement of GTI, some scholars use green patents to evaluate the level of GTI. For example, Du et al. [56] use the number of green technology patents as the proxy variable of the level of GTI to study the impact of environmental regulations at different levels of economic development on GTI. Some authors use parametric analysis to measure the level of GTI. For example, Lv et al. [57] measure the green total factor productivity (GTFP) of 30 provinces in China from 2003 to 2017 and find that the financial structure is conducive to GTI, while the financial scale and financial efficiency have a negative impact on GTI. Wang et al. [17] use the GTFP decomposition index to refer to the level of GTI, and find that GTI has a positive impact on the region’s GTFP and a negative impact on the periphery. As an important part of green innovation [58], GTI can effectively alleviate the pressure on resources and the environment in the process of promoting economic modernization [59]. To solve the current severe climate change and environmental pollution issues, it is of great significance to study the impact of GTI on CDE.

When we consider the regional heterogeneity and spatial interconnection, traditional econometric models are inefficient [60]. Spatial econometric models consider the spatial dependence between observations, and different approaches are used when spatial effects are considered [61]. Among them, the spatial autoregressive model (SAR), the spatial error model (SEM), and the spatial Durbin model (SDM) are the three most commonly used spatial econometric models [62]. Chen et al. [26] use the SDM to examine the influencing factors of urban eco-efficiency associated with technological innovations, and find that high innovative ability can increase urban eco-efficiency. Zhang et al. [63] use the SDM to examine the impact of high-speed rail on the consumption of urban residents, and found that HSR had different effects on consumption density in cities in eastern, central, and western China due to different levels of urban economic development and inter-city relationships. Yao et al. [60] use the SAR, SEM, and SDM to analyze the impact mechanisms of urbanization dimensions and the internal structure effect of each dimension on eco-efficiency, and find that population and ecological urbanization have a significant positive impact on local eco-efficiency, while social and spatial urbanization have a negative impact. Chica-Olmo et al. [64] use the SAR, SEM, and SDM to investigate the spatial dependence between GDP and renewable energy consumption for 26 European countries. Considering the existence of spatial interaction, it is of great significance to analyze the spatial spillover effect of GTI on CDE.

The existence of regional heterogeneity often lead to different results of innovation. For example, in some regions, despite favorable conditions and successful innovation factors, many attempts have failed, while in other regions, such attempts have been successful [65]. This phenomenon can be explained by innovative susceptibility of regions. Innovative susceptibility is an economic phenomenon relevant to certain level of scientific and technological progress and market relations [66]. Regional innovation sensitivity refers to the availability and ability of regional units to create and apply innovation based on existing conditions and resources in a specific and continuous regional innovation policy. Belyakova et al. [66] discuss the issues related to the content of the definitions “innovative susceptibility” and “regional innovation system”, and reveal the characteristics and classification of innovative susceptibility of the region. Volkova et al. [67] regard the innovation susceptibility of the regional economy as a condition of the digitalization of the regional economy, and propose an evaluation method of the innovation susceptibility to clarify the influence of various factors on the innovation susceptibility of the region and the pace of digital transformation. From the literature review, we can conclude that different regions differ in the degree of innovative development, owing to the innovative susceptibility. Therefore, it is necessary to conduct an in-depth analysis of the regional heterogeneity of GTI.

In the literature, there is a bulk of research on the relationship between technological innovation and carbon emissions. These studies have provided us with important references and enlightenment, but there are still some deficits. First, existing research has investigated the relationship between carbon emissions and technological innovation [68,69,70,71], but little on the GTI, which is more relevant to carbon emissions. Second, existing research on GTI focuses more on the overall research at the national level in China, but little on the regional heterogeneity of GTI, which results in a fragmentary understanding of the regional heterogeneity of GTI [72,73,74]. Last, the analyses of the impact of GTI on environmental issues in the literature do not consider spatial correlation [73,75,76,77]. Considering that China is a country with significant regional differences in resource endowments, economic structures and policy environments, ignoring spatial spillover effects will inevitably lead to biased results.

The contributions of this study are threefold. First, this paper combines the SBM (Slack Based Measure) model and the GML (Global Malmquist-Luenberger) index to measure GTI by constructing an environmental pollution unexpected output, and uses the Gini coefficient to measure the regional differences of GTI. This is conducive to a clear and comprehensive understanding of the phase characteristics of China’s green technology and its regional development characteristics, and lays the foundation for subsequent research on GTI. Second, it uses the kernel density estimation method to reveal the spatiotemporal evolution characteristics of carbon emissions, which helps us to obtain a clearer understanding of the spatial distribution and evolution characteristics of CDE. Third, it focuses on the spatial spillover effects of GTI on carbon emissions, through which the spatial correlation of CDE is fully considered and the research settings are therefore more realistic.

This paper uses the SBM model and GML index to measure the GTI of China’s 30 provincial administrative regions from 2001 to 2019. It constructs a spatial panel model to analyze the spatial spillover effects and nonlinear effects of GTI on carbon emissions. Regardless of the analysis of the regional spatial distribution characteristics of GTI and CDE, or the study of GTI’s spatial spillover effects of CDE, it is an enrichment and improvement of the theoretical system of carbon emission reduction in environmental economics. This study can also help policy makers to fully consider the spatial spillover and nonlinear impact of GTI on carbon emissions, and formulate different regional policies and measures based on the development characteristics of each province.

## 3. Materials and Methods

### 3.1. The Measurement Method of Green Technology Innovation

Data envelopment analysis (DEA) is a method for evaluating the relative effectiveness of decision-making units with multiple inputs and multiple outputs. It does not need to set a specific function form, which can effectively avoid the negative effects of subjectively setting the production function. However, the traditional DEA model does not consider the influence of the “slack variable” on the efficiency value. In order to solve this problem, Tone [78] proposed the Slack-Based Measure (SBM) model based on input and output slack variables. The SBM model addresses the problem of slack variables and is a good solution to the problem of green technology efficiency evaluation in which undesired outputs are included [11].

The total factor productivity is an important indicator to measure efficiency growth. Chung et al. [79] propose to apply the directional distance function, which contains undesired outputs to the Malmquist model, and call the Malmquist index as the Malmquist-Luenberger (ML) index. The ML index is often used to measure the growth of GTFP [80], but the index in the form of geometric average cannot observe the long-term growth trend of production efficiency, and the mixed directional distance function can also easily lead to infeasible solutions for linear programming. For this reason, Oh [81] constructed the Global Malmquist-Luenberger (GML) index on the basis of the ML index. The expression of the GML index is as follows:

(1) $$GML_{t}^{t+1}=\frac{1+{\vec{D}}_{0}^{G}(x^{t},y^{t},b^{t};y^{t},-b^{t})}{1+{\vec{D}}_{0}^{G}(x^{t+1},y^{t+1},b^{t+1};y^{t+1},-b^{t+1})}=EC_{t}^{t+1}\times TC_{t}^{t+1}$$

where $x\in R^{+}$ is the input vector; $y\in R^{+}$ is the desired output vector; and $b\in R^{+}$ is the undesired output vector. The input-output value in period t can be expressed as $\left(x^{t},y^{t},b^{t}\right)$. ${\vec{D}}_{0}{}^{G}(x^{t},y^{t},b^{t};g)=\sup\left\{\beta :(y,b)+\theta g\in P(x)\right\}$ is the global directional distance function of period t, $g=(g_{y}-g_{b})$ is the directional deviation function of desired output and non-desired output; *θ* denotes the maximum multiple of desired output and non-desired output that can be increased along the directional vector *g* with given inputs.

Since the evaluated decision unit is included in the global reference set, the GML index always has a feasible solution. $GML_{t}^{t+1}$ can be decomposed into technical efficiency change index ($EC_{t}^{t+1}$) and technical progress change index ($TC_{t}^{t+1}$), which represent the degree of technical efficiency improvement and technical progress of the decision-making unit DMU from t to t+1 period, respectively. The input of GTI in this article includes energy consumption, capital, and manpower, and the output level includes not only expected outputs, but also unexpected outputs. Therefore, this article measures China’s GTI based on the SBM model and GML index [17,57].

### 3.2. Carbon Emissions Estimation

Since China’s existing statistics do not include data on carbon emissions in various provinces, we estimate carbon emissions as the product of the consumption of various energy sources and the corresponding carbon emission coefficients, which is a common method to measure carbon emissions [9,11,82,83,84,85,86,87]. Based on the energy consumption data of the China Energy Statistical Yearbook and the practices of Wei and Li [88] and Liang et al. [89], we select 14 types of fossil fuels to calculate the CDE:

(2) $$CE=\sum_{i=1}^{14}CO_{2,i}=\sum_{i=1}^{14}E_{i}\times NCV_{i}\times CEF_{i}$$

where *CE* means CDE, $E_{i}$, $NCV_{i}$, and $CEF_{i}$ represent the consumption, the average net calorific value, and the carbon dioxide emission factor of energy fuel i, respectively. The fourteen types of fuels are energy fuels, namely, coal, coke, coke oven gas, blast furnace gas, converter gas, other gas, crude oil, gasoline, kerosene, diesel, fuel oil, liquefied petroleum gas, natural gas, and liquefied natural gas. The calculation formula is $CEF_{i}=CC_{i}\times COF_{i}\times 44/12$, where $CC_{i}$ and $COF_{i}$ are the carbon content and the carbon oxidation factor of fuel *i*, respectively. The constant 44/12 is the molecular weight ratio of carbon dioxide to carbon.

Carbon emission (*CE*) is often closely related to economic activities. It is natural that a rapidly developing economic body may cause large-scale carbon emissions. As such, to reduce carbon dioxide emissions may harm economic development. Effectively reducing carbon dioxide emissions and meanwhile achieving economic development are of vital importance. We often use the carbon emission intensity (*CI*) indicator to reflect the degree of the dependence of economic development on carbon. Reducing the intensity of carbon emissions is the key to solving the current conflicts among economic development, energy constraints, and environmental pressures. The *CI* index is expressed as:

(3) CI=CE/GDP

### 3.3. Models

Ordinary least squares (OLS) is the classical method used to estimate panel data models and assumes independence between observations [60]. However, everything is related to everything else, but near things are more related than distant things [90]. Compared with the traditional econometric model, the spatial econometric model takes into account the prevalent spatial dependence in economics, which is reflected in the lag term of the dependent variables and the error term in the spatial econometric model. The SAR is mainly used to study the direct spillover effects of the behavior of neighboring regions on the other regions in the entire system, and its spatial dependence is reflected in the lag term of the dependent variable. The SEM is mainly used to study the interaction between regions. The relationship is realized through the structural correlation of the error term, and its spatial dependence is reflected in the lag term of the error term. However, the transmission of spatial effects may occur simultaneously from spatial lags in the dependent variable and from changes in the error term caused by random shocks. To solve this issue, the SDM can be used and it takes into account not only the above two types of spatial transmission mechanisms, but also the spatial interaction. The OLS, SAR, SEM, and SDM model expressions are expressed as:

(4) $$CE_{it}=\beta_{1}GTI_{it}\;+\beta_{2}STR_{it}+\beta_{3}URB_{it}+\beta_{4}EDU_{it}+\beta_{5}\ln PGDP_{it}+\beta_{6}\ln TRA_{it}+\mu_{i}+\varepsilon_{it}$$

(5) $$CE_{it}=\gamma W_{0}CE_{it}+\beta_{1}GTI_{it}+\beta_{2}STR_{it}+\beta_{3}URB_{it}+\beta_{4}EDU_{it}+\beta_{5}\ln PGDP_{it}+\beta_{6}\ln TRA_{it}+\mu_{i}+\delta_{t}+\varepsilon_{it}$$

(6) $$\begin{array}{c} CE_{it}=\beta_{1}GTI_{it}+\beta_{2}STR_{it}+\beta_{3}URB_{it}+\beta_{4}EDU_{it}+\beta_{5}\ln PGDP_{it}+\beta_{6}\ln TRA_{it}+\mu_{i}+\delta_{t}+\varphi_{it}\\ \varphi_{it}=\tau W_{7}\varphi_{it}+\varepsilon_{it}\;\;,\;\;\varepsilon_{it}∼N\left(0,\;\sigma^{2}I\right) \end{array}$$

(7) $$\begin{array}{l} CE_{it}=\gamma W_{0}CE_{it}\;+\beta_{1}GTI_{it}+\beta_{2}STR_{it}+\beta_{3}URB_{it}+\beta_{4}EDU_{it}+\beta_{5}\ln PGDP_{it}+\beta_{6}\ln TRA_{it}+\theta_{1}W_{1}GTI_{it}\\ +\theta_{2}W_{2}STR_{it}+\theta_{3}W_{3}URB_{it}+\theta_{4}W_{4}EDU_{it}+\theta_{5}W_{5}\ln PGDP_{it}+\theta_{6}W_{6}\ln TRA_{it}+\mu_{i}+\delta_{t}+\varepsilon_{it} \end{array}$$

respectively, where $CE_{it}$ represents the CDE of province i in year t, $GTI_{it}$ represents the level of GTI in province i in year t, $STR_{it}$, $URG_{it}$, $EDU_{it}$, $PGDP_{it}$, and $TRA_{it}$ are the control variable vectors for separating other influencing variables, $W_{0},W_{1},\ldots ,W_{7}$ are the spatial weight matrixes, $\mu_{i}$ is the spatial individual effect, $\delta_{t}$ is the time effect, $\varphi_{it}$ is the error term, $\varepsilon_{it}$ is the stochastic error term, and γ, τ, $\beta_{1},\ldots ,\beta_{6}$, $\theta_{1},\ldots ,\theta_{6}$ are the corresponding regression coefficients, respectively. In order to ensure the reliability and robustness of the regression results, this paper will use SAR, SEM, and SDM models to perform regression analysis on the spatial spillover effects of GTI and carbon emissions.

In economic relationships, the phenomenon that an economic parameter reaches a certain value and causes another economic parameter to suddenly shift to other forms of development (structural mutation) is referred to as the threshold effect. The non-linear threshold effect cannot be described by ordinary linear regression, but can be done by threshold regression. Xie et al. [91] find that the relationship between energy consumption transition and green total factor productivity (GTFP) is inverse “N” type of nonlinear relationship through the threshold regression method. Du et al. [43] find that the impact of GTI on carbon emissions has a non-linear effect. Therefore, to further study whether the impact of GTI on carbon emissions depends on the level of regional economic development, we use Hansen’s panel threshold model [92]. This panel threshold model aims to incorporate a certain threshold value and empirically test and estimate the corresponding threshold value and the effect of the threshold [93]. The single-threshold model is as follows:

(8) $$CE_{it}=\beta_{1}\cdot \left[GTI_{it}\times I(s_{it}\leq q)\right]+\beta_{2}\cdot [GTI_{it}\times I(s_{it}>q)]+\beta_{c}X_{it}\;+\mu_{i}+\delta_{t}+\varepsilon_{it}$$

where $X_{it}$ is the control variable vector used to separate other influencing variables; $s_{it}$ is the threshold variable to represent the level of regional economic development, and q is the threshold parameter. For group $s_{it}\leq q$, the marginal effect of GTI is $\beta_{1}$, and for group $q_{it}>q$, it is $\beta_{2}$. More generally, a model with K thresholds can be expressed as follows:

(9) $$CE_{it}=\beta \cdot [GTI_{it}\times I(s_{it})]+\beta_{c}X_{it}\;+\mu_{i}+\delta_{t}+\varepsilon_{it}$$

where β is a *K*-dimensional vector, K is the number of thresholds, I(⋅) is an indicator function vector, and its kth component can be expressed as follows:

(10) $$I_{k}(s_{it})=\left\{\begin{array}{l} 1\;,\;q_{k-1}<s_{it}\leq q_{k}\\ 0\;,\;others \end{array}\;\right.$$

where $k\in \left\{1,\ldots ,K+1\right\}$, $q_{1}<q_{2}<\ldots <q_{K}$ are the K estimated threshold parameters, and $q_{0}\in (-\infty ,q_{1})$, $q_{K+1}\in (q_{K},+\infty )$.

### 3.4. Variables and Data Sources

To explore the relationship between GTI and carbon emissions, we select a sample of 570 observations from 30 provincial administrative regions in mainland China from 2001 to 2019 as an empirical study. Unless otherwise specified, the data in this work are taken from the China Statistical Yearbook, China Energy Statistical Yearbook, and statistical yearbooks of various provinces.

In this paper, CDE (CE) is used as the explained variable, which is calculated based on the energy consumption data of the China Energy Statistical Yearbook. We take GTI (GTI) as the core explanatory variable, follows the idea of Lv et al. [57] and Yang and Yang [94], measures GTI with green total factor productivity. Similar to Lv et al. [57], Fan and Xiao [16] and Xie et al. [91], we use capital stock, labor productivity, and energy consumption as inputs, regional GDP as expected outputs, and industrial waste gas, industrial wastewater, and industrial solid waste as undesired outputs (see Table 1). Finally, we use the SBM model and the GML index [17,91] to measure the level of GTI.

The control variables include

(1) the regional economic development level (PGDP), expressed by per capita GDP and calculated at constant prices based on the year 2000;
(2) industrial structure rationalization (STR), calculated by the reciprocal of the Theil index, which is measured by the number of employees and the ratio of output value among the three industries, and the expression is $TL=\sum_{i=1}^{n}\left(\frac{Y_{i}}{Y})\ln(\frac{Y_{i}/L_{i}}{Y/L}\right)$, where $Y_{i}$ represents the output value of industry i, $L_{i}$ represents the number of employees in the industry i;
(3) urbanization level (URB), calculated by the proportion of urban population;
(4) education level (EDU), measured by the average years of education in each province and calculated by the formula: [(primary school population × 6 years) + (junior middle school population × 9 years) + (senior high school population × 12 years) + (junior college population and above × 16 years)]/(population aged 6 years and above);
(5) openness (TRA), expressed in terms of total import and export volume at the location of the business unit. Table 2 gives the statistical description of the relevant variables.

## 4. Results

### 4.1. Evaluation of Green Technology Innovation Indicators

#### 4.1.1. The Estimation Results of GTI

**Figure 1:**
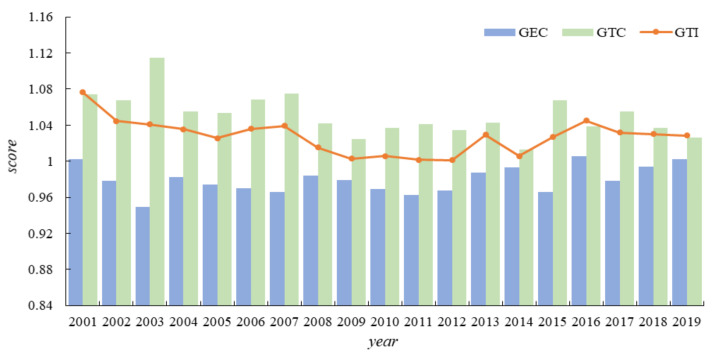
GTI and its decomposition, 2001–2019.

China’s GTI was estimated using Equation (1). The results shown in Figure 1 illustrate that from 2001 to 2019, the overall development of GTI was stable and the score remained above 1, which indicates that China’s GTI has achieved a positive growth in all years of the sample period. In the long run, China’s GTI level is in the range of 1.0014 to 1.0764, with an average level of 1.0275, showing a long-term stable development trend. In the short term, China’s GTI shows the development characteristics of local fluctuations, for example, the level of GTI was 1.0014 in 2012, increased to 1.0295 in 2013, decreased to 1.0058 in 2014, and then increased to 1.0449 in 2015. This suggests that the innovation speed is constantly changing, but the level of GTI is constantly improving, which is also evident in other empirical studies [17]. Through the GML index, GTI can be decomposed into green technological change (GTC) and green technical efficiency change (GEC). The GTC reflects the extent to which the frontier has been moved. ECH is the degree of change in the green technical efficiency of the DMU from period t to period t+1. It shows from Figure 1 that the GTC ranges from 1.0126 to 1.1149, with an average level of 1.0510, indicating that China has achieved a positive green technology progress every year and the rate of progress is higher than that of GTI. This is because GEC is generally not high. The innovation efficiency ranges from 0.9490 to 1.0060, with an average value of 0.9795, and the index level is generally below 1. This suggests that China only pays attention to the extent of technological progress, but ignores the importance of innovation efficiency, which results in that the overall level of China’s GTI is not high. This conclusion is also confirmed in other empirical studies [17].

#### 4.1.2. Regional Heterogeneity of GTI

Dagum [95] proposes the Gini coefficient method that sets the overall Gini coefficient (G) to be: $G=G_{w}+G_{nb}+G_{t}$, where $G_{w}$ represents the distribution differences in the GTI between provinces within a region; $G_{nb}$ represents the distribution differences in the GTI between provinces in different regions; and $G_{t}$ represents the remainder of the Gini coefficient due to the cross-impact of the GTI between different regions [96,97].

Through the Gini coefficient method, we measured and decomposed the regional differences in the GTI in China (see Table 3), which helps us to obtain a clearer understanding of the spatial distribution and evolution characteristics of GTI. This is also one of the contributions of this article.

**Figure 2:**
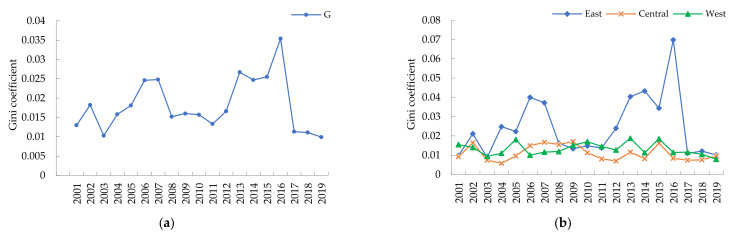

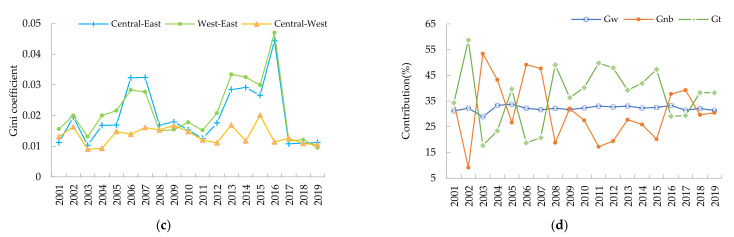
The trends of the Dagum Gini coefficient of GTI in China. (**a**) The overall Gini coefficient of the GTI in China. (**b**) The intra-regional differences of the GTI. (**c**) The inter-regional differences of the GTI. (**d**) The evolution of the contribution rate.

Figure 2 reveals the law of regionally differentiated development of China’s GTI. Figure 2a describes the trends of the overall Gini coefficient of China’s CTI. It illustrates that from 2001 to 2019, the overall Gini coefficient of China’s GTI has a stable long-term trend, but large short-term fluctuations. During the sample period, the overall Gini coefficient of GTI fluctuates between 0.0099 and 0.0354, around the average value 0.0182 and decreases by 1.50% annually. Figure 2b shows the intra-regional differences and their evolution of the GTI in the eastern, central, and western regions. Overall, the intra-regional difference of the eastern region displays a volatile development law, the central and western regions show a steady development trend in intra-regional difference. Figure 2c describes the inter-regional differences in the eastern, central, and western regions and their trends. The figure shows that the inter-regional Gini coefficients of the eastern and central regions and the eastern and western regions have relatively consistent trends, with inter-regional Gini coefficients fluctuating between 0.0103~0.0444 and 0.0095~0.0470, respectively. It shows significant fluctuations, while the inter-regional Gini coefficients in the central and western regions fluctuate between 0.0090~0.0201 show a more stable trend. Figure 2d illustrates the sources of the differences in China’s GTI and the trends of their contributions. It shows that the mean contributions of the intra-regional differences, inter-regional differences, and intensity of transvariation towards regional differences in China’s GTI during the study period are 32.15, 31.02, and 36.83%, respectively. From the evolution process, the contribution due to the intra-regional differences is relatively stable during the study period as it fluctuates within 28.89~33.74%. The contribution of the inter-regional differences and that from the intensity of transvariation experience a dynamic evolution process of repeated increases and decreases.

The above results indicate that China’s GTI has developed steadily for a long term, but fluctuates greatly in some years during the study period. In terms of the regional distribution, the imbalance among the eastern, central regions and the eastern, western regions is more prominent. Within the three major regions, the imbalance in the eastern region is also the most prominent. The possible reason is that the eastern coastal area is the most economically developed area, in which the infrastructure is well developed and the investment in scientific research is the strongest. Therefore, the level of GTI in the eastern region is better than the central and western regions [98]. However, not all provinces in the eastern region have highly developed economies, and some provinces are still sluggish in development and have a rigid industrial structure. Therefore, within the eastern region, there is a large gap in GTI among provinces.

### 4.2. Evolution Characteristics of the Carbon Emissions

#### 4.2.1. The Estimation Results of Carbon Emissions

**Figure 3:**
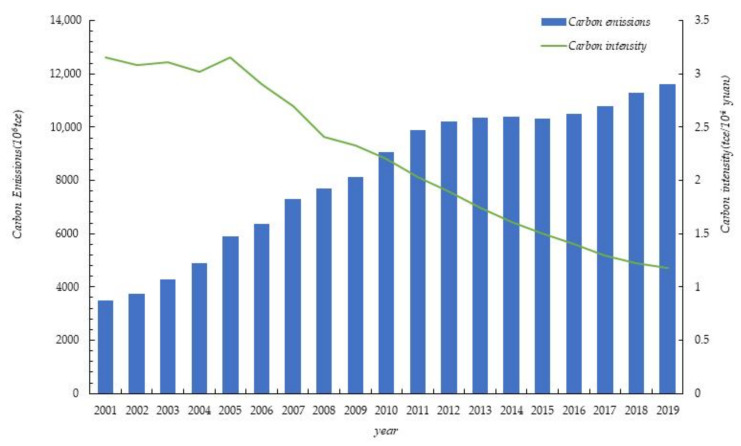
China’s carbon emissions and carbon emission intensity, 2001–2019.

This paper uses Equation (2) to estimate the CDE of various provinces in China from 2001 to 2019, and obtain the CDE intensity on this basis. Figure 3 depicts the average level of China’s carbon emissions and carbon emission intensity over the years. It can be found that CDE increased at a marginal declining rate from 2001 to 2019. From 2001 to 2011, carbon emissions increased significantly, with an average annual growth rate of 10.95%; after 2011, CDE have further slowed down, with an average annual growth of 1.87%. This suggests that China has achieved a staged victory in controlling the total amount of CDE, which is of great significance for gradually advancing the strategic goal of carbon peaking and carbon neutrality. We can also know from the figure that from 2001 to 2005, the carbon emission intensity fluctuated between 3.0195 and 3.1566, with an average annual decrease of 0.04%. The evolution of carbon emission intensity shows a characteristic of stable development. From 2006 to 2019, the carbon emission intensity fluctuated between 1.1782 and 2.9067, with an average annual decrease of 6.71%, and the carbon emission intensity showed a significant downward trend. This shows that, over time, the amount of carbon dioxide emitted per unit of GDP produced in China has declined significantly, and that China’s transition to a low-carbon economy, which used to rely on the traditional development model of high pollution and high emissions, has yielded significant results.

#### 4.2.2. Spatial Characteristics of Carbon Emissions

**Figure 4:**
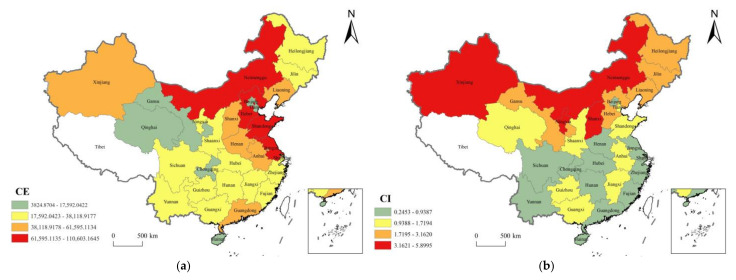
Spatial distribution of CE and CI in China in 2019. (**a**) Spatial distribution of CE. (**b**) Spatial distribution of CI. (Note: The graphics are drawn by ArcGIS software (Version 10.8) based on the results of CE and CI calculations).

A distinctive feature of China’s carbon emissions and carbon emission intensity is its temporal and spatial heterogeneity. Although China’s current growth rate of CDE is slowing down and the intensity of carbon emissions continues to decline, there is still a problem of regional imbalance in carbon emissions in various regions of China. This paper uses ArcGIS, an online geographic information system, to characterize the spatial distribution and intensity of carbon emissions in various regions of China in 2019. The results are shown in Figure 4. Figure 4a describes the CDE of various provinces in China and shows that there is a large gap in carbon emissions among provinces. Provinces Hebei, Shandong, Jiangsu, and Inner Mongolia are ranked in the first echelon in terms of carbon emissions, which are all maintained above 80 million tons; Provinces Tianjin, Gansu, Chongqing, Beijing, Qinghai, and Hainan are ranked in the fourth echelon in terms of carbon emissions. Carbon emissions are generally less than 200 million tons. As the province with the highest carbon emissions (110,603.16), Hebei is about 29 times more than Hainan (3824.87), the province with the lowest carbon emissions. From the perspective of regional distribution, the east and central regions are generally higher than the western regions. The possible reason is that the east and central regions of China have a complete industrial system, including a large number of heavy and light industries, while the industrial system in the western regions is relatively backward, resulting in more carbon emissions in the east and central regions than in the western regions. Figure 4b describes the carbon emission intensity of various provinces in China. As can be seen from the figure,

(1) the carbon emission intensities of provinces Ningxia, Inner Mongolia, Xinjiang, and Shanxi are ranked the first echelon, with their carbon emission intensity remaining above 3 tons/10,000 yuan;
(2) provinces Yunnan, Jiangsu, Henan, Hunan, Hubei, Hainan, Chongqing, Fujian, Sichuan, Zhejiang, Shanghai, Guangdong, and Beijing have carbon emission intensities below 1 ton/10,000 yuan, which belongs to the fourth echelon of carbon emissions.
(3) province Ningxia has the highest carbon emission intensity (5.8995) and is 24 times that of Beijing (0.2453), which has the lowest carbon emission intensity.

In terms of the regional distribution, the carbon emission intensity of the northern region is much higher than that of the southern region. The possible reason is that carbon emissions in the north are generally higher, and economic development is lagging behind that in the south, resulting in a generally higher average CDE required to produce a unit of GDP. In addition, residents in the north need energy to heat their homes in the cold weather and therefore generate more carbon emission.

**Figure 5:**
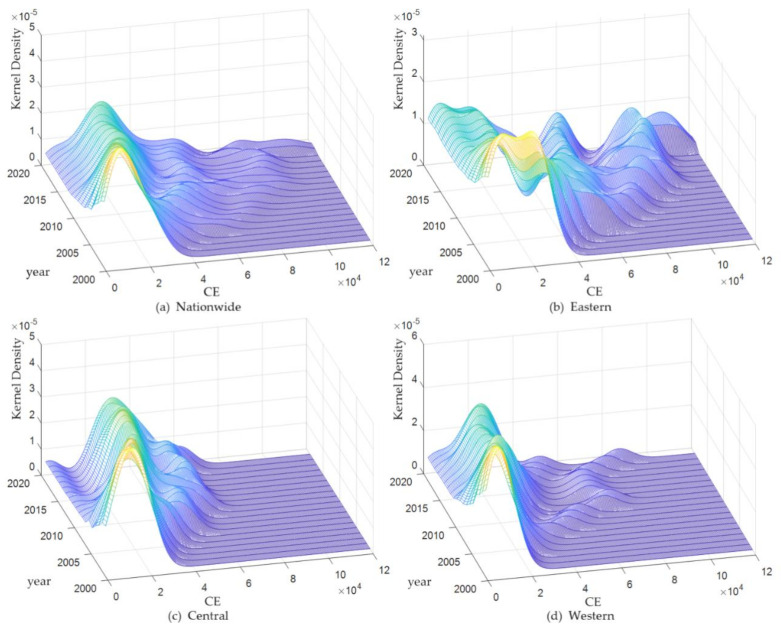
The dynamic evolution characteristics of CE in various regions, 2001–2019. Note: The graph is drawn by MATLAB software (version R2019b) based on the GTI calculation result.

Based on the analysis of the regional heterogeneity, we further analyze the characteristics of the temporal and spatial evolution of CDE. Figure 5 shows the dynamic evolution of CDE across the country and the eastern, central, and western regions from 2001 to 2019. Figure 5a reveals the characteristics of the dynamic evolution of national carbon emissions. From the perspective of the distribution position, the position of the main peak of the national carbon dioxide emission distribution curve moves slowly to the right, indicating that the country’s CDE are slowly increasing. In terms of the distribution pattern of the main peak, the peak of the main peak of the national carbon emission density function is increasing and the width of the main peak is decreasing, indicating that the absolute difference in national carbon emissions is showing a decreasing trend. From the perspective of the number of peaks, the national carbon emissions in most years showed a multi-peak distribution, and the heights of the main peak and side peaks were quite different, indicating that the national carbon emissions distribution showed a weak multi-polarity trend. In terms of the extension of the distribution, the kernel density curve shows a trailing rightward trend and a widening of the extension of the distribution, which implies that there are provinces with higher CDE across the country, and this emission gap has a tendency to further expand. Figure 5b–d illustrates the dynamic evolution characteristics of carbon emissions in the eastern, central, and western regions, respectively. From the distribution position, the main peak of the distribution curve of carbon emissions in the eastern and western regions shows an overall rightward trend, indicating that CDE in the eastern and western regions have a tendency to increase, while the main peak in the central region first shifts to the right and then slowly shifts to the left, indicating that the central region is slowly decreasing after experiencing an increase in carbon emissions. From the perspective of the distribution pattern of the main peaks, the three regions show the characteristics that the peak of the main peak first declines and then rises or keeps rising, and the width of the main peak gradually decreases, indicating that the absolute difference of carbon emissions within the three regions shows a decreasing trend. In terms of the number of peaks, carbon emissions in the eastern region show a multi-peak distribution, with the main and side peaks being of equal height, and even the side peaks exceeding the main peak in height in some years. This implies that there is a significant multi-level differentiation in the eastern region, while the distribution of carbon emissions in the central and western regions shows a polarized difference.

### 4.3. Spatial Spillover and Nonlinear Effects

#### 4.3.1. Analysis of Spatial Spillover Effects

Due to the geographical proximity, economic activities between neighboring regions are closely related. As an undesired output of economic activities, carbon dioxide will also proceed simultaneously and evolve in coordination. Therefore, to explore the impact of GTI on carbon emissions, it is necessary to consider whether the technological innovation of a certain province will have a significant impact on the carbon emissions of neighboring provinces. To further test the spatial effects of GTI on carbon emissions, we will use the spatial panel measurement model to analyze the relationship between them. At the same time, to ensure the reliability of the test results, we use the adjacent weight matrix (w1) and geographic distance weight matrix (w2) to measure the performance of the space model.

An element in the adjacency weight matrix is the value to indicate the degree of the proximity: it is 1 if two provinces are adjacent, and 0 otherwise. An element in the geographical distance weight matrix measures the reciprocal of the distance between provinces. In order to judge whether regional carbon emissions can be statistically analyzed through spatial measurement, it is necessary to examine whether the variables are spatially correlated. Moran’s I index reflects the degree of similarity between spatially adjacent or spatially adjacent regional unit attribute values [99], and is usually used to test the spatial correlation of variables. The value range of Moran’s I index is [−1, 1], a value greater than 0 suggests a positive correlation, a value 0 suggests no correlation, and a value less than 0 suggests negative correlation. The results of this paper on the global Moran’s I index of carbon emissions are shown in Table 4. It can be found that the global Moran’s I index of China’s carbon emissions from 2001 to 2019 is between [0.2400, 0.3060] and [0.2040, 0.2930] under the two weights. They all passed the significance test, indicating that there is a significant positive spatial correlation between carbon emissions.

**Figure 6:**
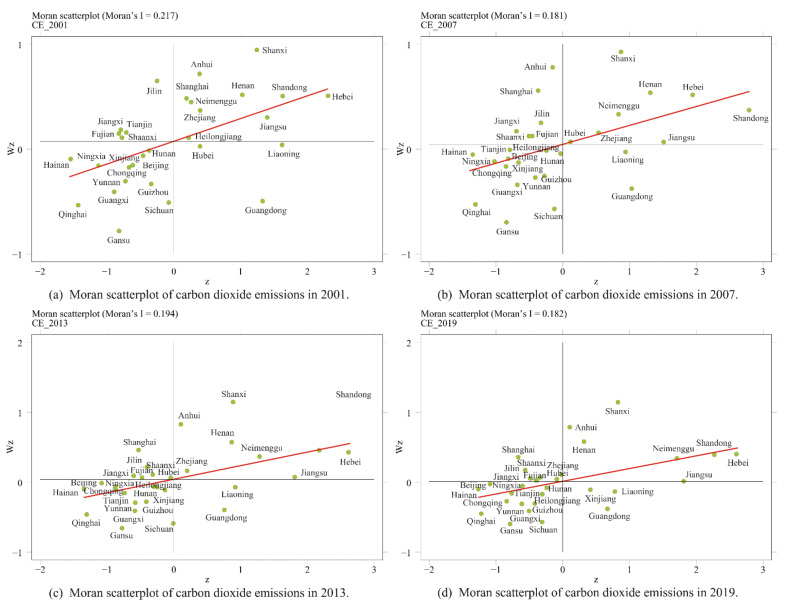
Moran scatterplot of carbon dioxide emissions.

Since the global spatial autocorrelation focuses on describing whether there is spatial agglomeration of variables in the overall distribution space, ignoring the issue of spatial correlation between regions, the spatial heterogeneity characteristics need further investigating with Moran’s scatterplot. By testing the global spatial correlation, we draw Moran scatter plots in 2001, 2007, 2013, and 2019 to further analyze the spatial agglomeration of carbon emissions, respectively. In a Moran’s scatterplots, the region under examination is divided into four parts, namely H-H, L-H, L-L, and H-L, which in turn correspond to quadrants I, II, III, and IV of the scatter plot, respectively. The first quadrant indicates that these provinces have high values, and the neighbor provinces have high values; the second quadrant indicates that these provinces have low values and the neighbor provinces have high values; the third quadrant indicates that these provinces and neighbor provinces have low values; the fourth quadrant indicates that these provinces have high values, and the surrounding provinces have low values. When provinces are of H-H type or L-L type, it suggests that the provinces have positive spatial autocorrelation. If provinces are of L-H type or H-L type, it suggests that the provinces have negative spatial autocorrelation. Figure 6 shows Moran’s scatter plot based on the geographical distance weight matrix (w2). It shows that most of the provinces in different years fell into the first and third quadrants, in the H-H type agglomeration area or L-L type agglomeration area. This indicates that carbon emissions show positive agglomeration characteristics. From 2001 to 2019, the scattered points gradually moved from the second and fourth quadrants to the first and third quadrants, indicating that the characteristics of positive carbon emission accumulation have been continuously strengthened. Provinces Hebei, Shanxi, Neimenggu, Jiangsu, Shandong, Henan always fall in the first quadrant, while province Beijing, Hunan, Guangxi, Hainan, Chongqing, Sichuan, Guizhou, Yunnan, Gansu, Beijing, Hunan, Guangxi, Hainan, Chongqing, Sichuan, Guizhou, Yunnan, Gansu, Qinghai, Ningxia always fall in the third quadrant, indicating that the spatial correlation of carbon emissions has a strong stability.

The results of the above spatial correlation test show that there is a positive and long-term stable global correlation in carbon emissions in various regions of China, and the local spatial correlation shows positive agglomeration characteristics. We will then use the spatial measurement method to build a regression model to examine the impact of GTI on CDE. In order to ensure the reliability and robustness of the regression results, we use the SDM, the SEM, and the SAR to perform regression analysis, respectively.

Table 5 depicts the regression results of the impact of GTI on carbon emissions, where the regression results under column (1) is ordinary least squares (OLS). It shows that the coefficient of the impact of GTI on CE is −0.5570 and significant at the 1% significance level, suggesting that on average, increasing every 1% in the level of GTI can effectively contribute to a reduction of carbon emissions by 0.557%. Columns (2) and (3) are the regression results of the SDM model under the weight matrices of w1 and w2, respectively. It can be found that the coefficients of the effect of GTI on carbon emissions are −0.3449 and −0.3761, both of which are significant at the 10 and 5% significance levels, respectively. Similarly, the regression results of the SEM and SAR models under the weight matrices w1 and w2 can be obtained. The influence coefficients of GTI on GE are −0.3999, −0.5539, and −0.4245, −0.5597, respectively. All results are negative and significant at the 5% significance level. The four sets of estimates show that the coefficient of impact of GTI on carbon emissions remains between −0.5597 and −0.3449, indicating that on average, increasing every 1% in the level of GTI results in a reduction of at least 0.3449% in carbon emissions. From the perspective of measurement methods, Table 5 compares OLS estimates with SDM, SEM, and SAR estimates. From the table, we can see the fit of the four models through AIC (Akaike information criterion) [100] and BIC (Akaike information criterion) [101], both of which are estimators of prediction error of statistical models [102]. As such, the model with the smallest AIC or BIC is preferred. The spatial ρ is the intensity of the spatial interdependency. Take the calculation results of the SDM model and the adjacent weight matrix (w1) as an example, it explains that 1% rise in adjacent carbon emissions is expected to be associated with a 0.4188% rise in local carbon emissions while the other variables are hold unchanged. λ is an autoregressive parameter that traces the spatial effect in error term. Table 5 indicates that local carbon emissions increased by 0.4539% with 1% increase in neighbor carbon emissions. This means that carbon emissions relate to space and transmit from one region to the other country, which creates the uncertainty of its growth process. In conclusion, the results of the four sets of estimates are relatively robust and the differences in measurement methods do not lead to significant differences in the results, which strongly suggests that GTI has a significant contribution to the reduction of carbon emissions.

According to the scope and object of the spatial effect, the effect of independent variables on dependent variables in spatial measurement models can be divided into direct effects, indirect effects, and total effects [17]. The direct effect reflects the impact of GTI on carbon emissions in the region, the indirect effect reflects the impact of GTI on carbon emissions in other regions, and the total effect reflects the average impact of GTI on carbon emissions in the entire region, with the results shown in Table 6. Under the SDM model, the direct and indirect effects of GTI on carbon emissions are −0.3945 and −0.7878, respectively, which are significant at the level of 1 and 10%. The indirect effect is greater than the direct effect, indicating that GTI has significant spatial spillover effect. Improving the level of local GTI can not only reduce CDE in the region, but also promote carbon emissions reduction in neighboring regions. The results under the SAR model are similar, with a significant direct and indirect effect of GTI on carbon emissions. The total effects under the two models are both negative and significant at the level of 5%, indicating that China’s GTI has a significant role in reducing CDE, and this positive impact has a significant spatial spillover effect.

#### 4.3.2. Nonlinear Effect Analysis

Some previous studies suggest that the effect of GTI on CDE can be positive or negative under different conditions [43]. We argue that the existence of differences in the level of regional economic development is an important factor relating to the non-linear relationship between GTI and CDE. This is because: when the economic development is relatively backward, less investment on GTI will be made and the region may therefore adopt a traditional model with greater pollution to promote a rapid development of the regional economy; as a result, GTI’s effect on reducing CDE is relatively weak. On the other hand, when the economy is highly developing, sufficient R&D investment will be made and the effect of GTI on reducing CDE will therefore increase significantly.

To test the above presumption and investigate the non-linear relationship between GTI and carbon emissions, we take the development level of each regional economy as the threshold, and establish a single threshold, a dual threshold, and a triple threshold model. The threshold value was sampled for 400 times. The final results show that there is a single threshold between GTI and carbon emissions. The specific results are shown in Table 7. It can be seen from the table that when using the level of economic development as the threshold variable, the results of the threshold regression model show that this threshold is 9.6509. When the economic development level is below 9.6509, the impact coefficient of GTI on carbon emissions is −0.9096, which is significant at the level of 1% and indicates that when the economy is at a low level of development, on average, increasing every 1% in GTI can reduce CO2 emissions by 0.9096%. When the development level of the economy is above 9.6509, the impact coefficient of GTI on CDE becomes −0.6782, indicating that as the development level of the economy increases, the driving effect of GTI on carbon emission conservation decreases. The possible reason is that when the economy is highly developed, the marginal reduction in emissions from investing capital in GTI is diminishing, making the reduction in emissions from GTI more significant in areas that are relatively less developed.

#### 4.3.3. Robustness Test

To further verify the reliability of the regression, we incorporate regional economic activities into the construction of the weight matrix, and construct an economic distance weight matrix (w3) [103] that considers the economic correlation between regions. Economic factors play an important role in the connection of various elements between different regions. Therefore, it is of important practical significance to study the effect of GTI on the basis of economic distance on the spatial spillover of CDE. The economic distance weight matrix is expressed as

(11) $$w_{3}=\left\{\begin{array}{c} 1/\left|{\bar{y}}_{i}-{\bar{y}}_{j}\right|,i\neq j\\ \;\;\;0\;\;\;\;,i=j \end{array}\right.$$

where i and j represent provinces, and $\bar{y}$ represents the real GDP per capita of a province from 2008 to 2018.

Under the economic distance weight matrix, the spatial spillover effects of GTI on carbon emissions are multi-dimensionally measured, and the regression results are shown in Table 8, where column (1) is the regression result of the SDM model under the economic distance weight matrix, column (2) is the regression result of the SEM model under the economic distance weight matrix. The significance level is 1% level. Although the coefficients of the estimated results are different, their direction and significance level have not fundamentally changed, which shows that the research results are robust and reliable. At the same time, this paper uses the method of replacing the spatial measurement regression model to perform further robustness testing. Specifically, because the generalized spatial panel random effects model (GSPRE) assumes that all the explained variables will affect other regions through spatial interaction, which is consistent with the hypothesis of spatial spillover effects, this paper uses the GSPRE model to investigate the relationship between GTI and carbon emissions. The results in column (3) show that the impact coefficient of GTI on carbon emissions is significantly negative at the 1% confidence level. The direction and significance level of the regression coefficients do not change fundamentally, compared to the previous spatial econometric regression. Therefore, the results of this paper are robust and reliable.

## 5. Conclusions

This paper studied the impact of green technology innovation (GTI) on carbon emissions, selected relevant panel data from 2001 to 2019 for 30 provincial regions in China, and comprehensively analyzed the spatial spillover and non-linear effects of GTI on carbon emissions. The main findings are the following.

First, China’s GTI has developed steadily and has achieved growth over the previous years. Among them, China’s green technology progress has improved significantly, but the innovation efficiency is not high, indicating that in the process of promoting GTI, China has paid too much attention to the extent of technological progress, but little to the importance of innovation efficiency. This not only causes the waste of human and material resources, but also leads to the low level of China’s overall green innovation.

Second, China’s total carbon dioxide emissions (CDE) have grown at a marginal rate of decline, and the intensity of carbon emissions has been declining year by year, indicating that China has achieved phased success in controlling CDE. However, at the current stage, China’s carbon emissions in various regions still have the regional development imbalances problem.

Third, GTI cannot only effectively reduce CDE in the region, but also have a significant spatial spillover effect on the reduction of CDE outside the region. From the perspective of the overall trend, as the development level of the economy increases, the effect of GTI on carbon emission reduction has weakened. A possible reason is that when the economy is highly developed, the marginal reduction in emissions from investing capital in GTI is diminishing.

Based on the above conclusions, our recommendations are as follows:

(1) In the process of increasing investment in GTI, each region should strive to improve the efficiency of technological innovation. At the same time, in view of the differences in regional economic development and carbon emissions, relevant regional policies and measures should be formulated in accordance with local conditions.
(2) From the perspective of the temporal and spatial distribution characteristics of China’s CDE, the government should coordinate regional development characteristics, accelerate the transformation and upgrading of the regional industrial structure, achieve coordinated planning for regional development, and promote coordinated regional development.
(3) In the process of formulating policies, local governments need to pay attention to both the production and development of the region, and the development strategies of the surrounding areas, and actively build a regional collaboration platform to promote the coordinated development of green multi-regional technological innovation and energy conservation and emission reduction.
(4) Taking into account the threshold characteristics of GTI, only by continuously increasing R&D expenditures on GTI and promoting the continuous development of GTI can regions effectively achieve the reduction of carbon dioxide and ensure the successful realization of carbon peak and carbon neutral goals.

Finally, it is worth pointing out that this paper attempted to provide new evidence on the heterogeneous impact of GTI on CDE, whereas measuring GTI is a challenging issue. Due to the data availability, we employed GTFP as the proxy of the GTI that has some potential limitations. Future research will aim to find ways to better measure GTI. Of course, the limitations do not cast doubt on the results of this study, but they should be addressed in future studies. In addition, in the study of regional differences, we can focus on the analysis of the innovative susceptibility of regions in the future. This can help us to obtain the sensitive differences in GTI from development to application in different regions, and play an important role in achieving regional joint reduction of carbon emissions.

## Figures and Tables

**Table 1:** The evaluation indicators of GTI.

Target Layer	First-Level Indicators		Second-Level Indicators	Index Definition
The evaluation indicators of green technology innovation	Input indicators		Capital stock	Capital stock based on 2000
			Labor force	Year-end employed persons
			Energy consumption	Total energy consumption
	Output indicator	Expected	Real GDP	Real GDP based on 2000
		Unexpected	Waste gas	Industrial waste gas emissions
			Wastewater	Total industrial wastewater discharge
			Solid waste	Industrial solid waste generation

**Table 2:** Statistical description of variables.

Type	Variable Name	Variable Declaration	Average Value	Standard Deviation	Min	Max
Explained variable	CE	Carbon Emission	27,404.3000	21,180.8200	856.4017	110,603.2000
Core explanatory variable	GTI	Green technology innovation	1.0275	0.0511	0.8200	1.6012
Threshold variable	PGDP	Economic level	34,416.2500	26,847.7800	3000.0000	164,563.0000
Control variables	STR	Industrial structure	0.0728	0.0846	0.0114	0.6192
	URB	Urbanization level	0.5169	0.1483	0.2035	0.9415
	EDU	Education level	8.6174	1.0517	6.0405	12.7820
	TRA	Foreign trade level	932.1860	1772.7710	1.9664	10,915.8100

**Table 3:** The Dagum Gini coefficient and its decomposition of GTI.

Year	*G*	Intra-Regional Differences			Inter-Regional Differences			Contributions (%)		
		East	Central	West	Central-East	West-East	Central-West	*G_w_*	*G_nb_*	*G_t_*
2001	0.0130	0.0098	0.0092	0.0155	0.0112	0.0156	0.0131	31.15	34.54	34.31
2002	0.0182	0.0211	0.0164	0.0140	0.0195	0.0200	0.0163	32.20	9.09	58.71
2003	0.0103	0.0088	0.0074	0.0094	0.0103	0.0131	0.0090	28.89	53.42	17.69
2004	0.0158	0.0248	0.0058	0.0110	0.0168	0.0200	0.0093	33.30	43.28	23.42
2005	0.0181	0.0223	0.0096	0.0181	0.0169	0.0216	0.0148	33.74	26.63	39.63
2006	0.0246	0.0400	0.0149	0.0100	0.0323	0.0283	0.0139	32.16	49.12	18.72
2007	0.0248	0.0372	0.0167	0.0116	0.0324	0.0277	0.0161	31.63	47.67	20.70
2008	0.0152	0.0162	0.0155	0.0119	0.0169	0.0150	0.0153	32.15	18.77	49.08
2009	0.0160	0.0134	0.0171	0.0151	0.0179	0.0154	0.0168	31.67	32.03	36.30
2010	0.0157	0.0148	0.0113	0.0170	0.0153	0.0178	0.0148	32.30	27.48	40.22
2011	0.0133	0.0138	0.0081	0.0145	0.0125	0.0152	0.0120	33.04	17.19	49.77
2012	0.0166	0.0240	0.0070	0.0127	0.0176	0.0208	0.0111	32.68	19.43	47.89
2013	0.0267	0.0404	0.0116	0.0188	0.0285	0.0334	0.0169	33.03	27.73	39.24
2014	0.0247	0.0433	0.0082	0.0113	0.0291	0.0325	0.0117	32.25	25.85	41.90
2015	0.0255	0.0344	0.0162	0.0185	0.0266	0.0299	0.0201	32.52	20.17	47.31
2016	0.0354	0.0698	0.0084	0.0114	0.0444	0.0470	0.0114	33.24	37.71	29.05
2017	0.0113	0.0110	0.0074	0.0116	0.0108	0.0118	0.0126	31.44	39.25	29.31
2018	0.0111	0.0121	0.0076	0.0105	0.0110	0.0121	0.0109	32.10	29.63	38.27
2019	0.0099	0.0101	0.0096	0.0079	0.0112	0.0095	0.0105	31.36	30.41	38.23

**Table 4:** Calculation results of the global Moran’s I values of CE, 2001–2019.

Year	w1			w2		
	I	z	*p*	I	z	*p*
2001	0.2940	2.9880	0.0010	0.2760	2.3150	0.0100
2002	0.2750	2.8260	0.0020	0.2930	2.4540	0.0070
2003	0.2530	2.6360	0.0040	0.2650	2.2550	0.0120
2004	0.2660	2.7600	0.0030	0.2460	2.1070	0.0180
2005	0.3060	3.1820	0.0010	0.2260	1.9920	0.0230
2006	0.2990	3.1060	0.0010	0.2070	1.8440	0.0330
2007	0.3060	3.1560	0.0010	0.2190	1.9230	0.0270
2008	0.3010	3.1130	0.0010	0.2250	1.9720	0.0240
2009	0.2910	3.0120	0.0010	0.2200	1.9280	0.0270
2010	0.2990	3.0860	0.0010	0.2080	1.8340	0.0330
2011	0.2760	2.8630	0.0020	0.2060	1.8170	0.0350
2012	0.2630	2.7540	0.0030	0.2040	1.8070	0.0350
2013	0.2860	2.9730	0.0010	0.2420	2.1010	0.0180
2014	0.2850	2.9560	0.0020	0.2330	2.0250	0.0210
2015	0.2810	2.9260	0.0020	0.2170	1.9160	0.0280
2016	0.2690	2.8160	0.0020	0.2220	1.9480	0.0260
2017	0.2400	2.5330	0.0060	0.2260	1.9700	0.0240
2018	0.2570	2.7220	0.0030	0.2340	2.0510	0.0200
2019	0.2400	2.5570	0.0050	0.2240	1.9710	0.0240

**Table 5:** Spatial panel regression results of GTI on CE.

EP	OLS	SDM		SEM		SAR	
	(1)	(2)	(3)	(4)	(5)	(6)	(7)
GTI	−0.5570 ***	−0.3449 *	−0.3761 **	−0.3999 **	−0.5539 ***	−0.4245 **	−0.5597 ***
	(0.1474)	(0.1358)	(0.1418)	(0.1357)	(0.1462)	(0.1376)	(0.1425)
STR	−0.5106 *	−0.4802 *	−0.3507	−0.5769 **	−0.4608 *	−0.6701 ***	−0.4639 *
	(0.1994)	(0.2113)	(0.1984)	(0.1914)	(0.1987)	(0.1858)	(0.1976)
URB	0.3828	1.0779 ***	1.1402 ***	1.0854 ***	0.7729 **	0.4844	0.3957
	(0.2794)	(0.2582)	(0.2700)	(0.2602)	(0.2987)	(0.2587)	(0.2704)
EDU	−0.1421 ***	−0.0883 **	−0.0931 **	−0.1192 ***	−0.1406 ***	−0.1317 ***	−0.1386 ***
	(0.0298)	(0.0296)	(0.0315)	(0.0292)	(0.0305)	(0.0276)	(0.0290)
lnPGDP	0.5706 ***	0.5838 ***	0.6215 ***	0.5271 ***	0.5705 ***	0.4467 ***	0.5739 ***
	(0.0478)	(0.0510)	(0.0457)	(0.0445)	(0.0468)	(0.0478)	(0.0463)
lnTRA	0.0460	−0.0042	−0.0218	0.0209	0.0190	0.0164	0.0526 *
	(0.0235)	(0.0238)	(0.0244)	(0.0229)	(0.0253)	(0.0221)	(0.0235)
ρ		0.4188 ***	2181.2510 **			0.2749 ***	−520.7427
		(0.0473)	(798.8768)			(0.0404)	(486.9011)
λ				0.4539 ***	2801.3080 **		
				(0.0465)	(892.9952)		
*n*	570	570	570	570	570	570	570
R^2^	0.8520	0.8598	0.8684	0.8488	0.8510	0.8485	0.8532
AIC	−492.9306	−580.5652	−558.2373	−566.0237	−500.0683	−533.8109	−492.0737
BIC	−462.5111	−519.7262	−497.3984	−531.2586	−465.3032	−499.0458	−457.3086

Note: ***, ** and * indicate that coefficients are statistically significant at 1, 5, and 10%, respectively. Standard errors are given in brackets. This table does not show variable lags.

**Table 6:** Spatial effect decomposition under SDM model and SAR model.

	SDM			SAR		
	Direct Effect	Indirect Effect	Total Effect	Direct Effect	Indirect Effect	Total Effect
GTI	−0.3945 ***	−0.7878 *	−1.1823 **	−0.4268 ***	−0.1446 ***	−0.5715 ***
	(0.1437)	(0.4207)	(0.4778)	(0.1435)	(0.0546)	(0.1915)
STR	−0.4983 ***	−0.1388	−0.6371	−0.6903 ***	−0.2374 ***	−0.9277 ***
	(0.1906)	(0.6417)	(0.6344)	(0.1831)	(0.0836)	(0.2560)
URB	0.8929 ***	−2.6883 ***	−1.7954 **	0.4851 *	0.1674 *	0.6525 *
	(0.2622)	(0.6870)	(0.8101)	(0.2613)	(0.0984)	(0.3542)
EDU	−0.0894 ***	−0.0070	−0.0965	−0.1350 ***	−0.0461 ***	−0.1810 ***
	(0.0279)	(0.0587)	(0.0652)	(0.0262)	(0.0125)	(0.0359)
lnPGDP	0.6119 ***	0.3307 ***	0.9426 ***	0.4597 ***	0.1560 ***	0.6157 ***
	(0.0487)	(0.1144)	(0.1279)	(0.0454)	(0.0285)	(0.0568)
lnTRA	−0.0123	−0.0968 *	−0.1090 *	0.0152	0.0049	0.0202
	(0.0225)	(0.0550)	(0.0610)	(0.0210)	(0.0071)	(0.0279)

Note: ***, ** and * indicate that coefficients are statistically significant at 1%, 5% and 10%, respectively. Standard errors are given in brackets.

**Table 7:** Regression results of the panel threshold of GTI on CE.

EP		Value	EP	Value	EP	Value
*p* value	Single	0.0010	GTI*I(Th < q)	−0.9096 ***	*n*	570
	Double	0.2133		(0.1548)	R^2^	0.8336
	Triple	0.6200	GTI*I(Th ≥ q)	−0.6782 ***	AIC	−426.2066
Threshold	q	9.6509		(0.1555)	BIC	−395.7871
Coefficient	STR	0.0396	URB	1.912 ***	CONS	8.5510 ***
		(0.2173)		(0.2473)		(0.2331)
	EDU	0.0363	lnTRA	0.1507 ***		
		(0.0273)		(0.0212)		

Note: *** indicate that coefficients are statistically significant at 10%, respectively. Standard errors are given in brackets.

**Table 8:** Spatial panel regression results of GTI on CE (robustness).

EP	SDM-w3	SEM-w3	GSPRE
	(1)	(2)	(3)
GTI	−0.6839 ***	−0.4910 ***	−0.5491 ***
	(0.1468)	(0.1424)	(0.1498)
STR	−0.7960 **	−0.5357 **	−0.5069 *
	(0.2659)	(0.2024)	(0.2028)
URB	0.6578 *	0.621 0*	0.6362 *
	(0.2714)	(0.2757)	(0.3029)
EDU	−0.1218 ***	−0.1358 ***	−0.1419 ***
	(0.0297)	(0.0308)	(0.0308)
lnPGDP	0.5984 ***	0.5994 ***	0.5630 ***
	(0.0494)	(0.0471)	(0.0471)
lnTRA	−0.0035	−0.0156	0.0367
	(0.0233)	(0.0245)	(0.0255)
ρ	0.2979 ***		
	(0.0081)		
λ		0.1083 ***	2436.4580 **
		(0.0125)	(926.9712)
*n*	570	570	570
R^2^	0.8412	0.8496	0.8517
AIC	−475.9781	−535.4121	−285.5882
BIC	−415.1392	−500.6470	−237.7862

Note: ***, ** and * indicate that coefficients are statistically significant at 1, 5, and 10%, respectively. Standard errors are given in brackets.

## Data Availability

Not applicable.
